# Clinical Aspects and Therapy of Sporadic Burkitt Lymphoma

**DOI:** 10.4084/MJHID.2009.030

**Published:** 2009-12-28

**Authors:** Livio Pagano, Morena Caira, Caterina Giovanna Valentini, Luana Fianchi

**Affiliations:** Istituto di Ematologia, Università Cattolica S. Cuore, Roma

## Abstract

Burkitt’s lymphoma is a highly aggressive mature B-cell neoplasm consisting of endemic, sporadic, and immunodeficiency-associated variants, sharing many morphologic and immunophenotypic features. It is characterized by a high proliferation rate and propensity for extranodal sites such as gastrointestinal tract and reproductive organs. Brief-duration, high-intensity chemotherapy regimens including aggressive central nervous system prophylaxis have had remarkable success in the treatment of this disease in the sporadic form, with very high complete remission rate and overall survival in adults. Although Burkitt’s lymphoma is extremely chemosensitive, biologically targeted therapies should be developed, because current treatment options are suboptimal for patients with poor prognostic features or with relapsed disease.

## Introduction:

Burkitt’s lymphoma (BL) is a small non-cleaved cell lymphoma with a high proliferation rate and characteristic molecular changes involving the *c-MYC* oncogene. It is a clinically distinct and aggressive disease, that frequently involves extranodal sites, such as the gastrointestinal tract and the central nervous system (CNS), so that it requires urgent treatment.

In the WHO classification three clinical variants are recognized: endemic, sporadic and immunodeficiency-associated 1. The three subtypes are identical according to histological pattern, and they all possess chromosomal rearrangements of the *c-MYC* oncogene, that contributes to lymphomagenesis altering the mechanisms of cell cycle regulation, cellular differentiation, apoptosis, cellular adhesion, and metabolism. BL is common in children, accounting for 40–50% of childhood non-Hodgkin’s lymphomas (NHL) in non-endemic areas 2,3.

These data have been recently update by a study about sporadic childhood BL incidence in United States during 1992–2005, reporting over this period 296 cases of children 0–14 years-old, accounting for approximately 30% of childhood NHL. The distribution of the cases indicated an early age onset (3–5 years) and a predominance in boys (79%) and in non-Hispanic Whites (81%), suggesting that male sex and factors correlated with race may be risk factors for sporadic BL 4.

These results were confirmed in another analysis conducted by the same Authors about age-specific incidence pattern for BL in US over the years 1973–2005. In this study a novel tri/bimodal incidence patterns for BL emerged, which showed disparities by gender but not race. In fact a notable finding was distinct trimodal age-specific BL incidence patterns among males, with three separate incidence peaks near ages 10, 40, and 75 years, respectively. Among females, the pediatric and the geriatric peaks were remarkable, but not the adult one. BL incidence rates were significantly higher among males for pediatric and adult BL, but marginally for geriatric patients^5^.

BL also occurs in adults, with the sporadic form accounting for 1–2% of all adult NHL in the western Europe and in the United States^6^.

## Clinical Aspects:

Unique clinical features have been described among the 3 different variants of BL, although there is considerable overlap among them.

The sporadic form is mostly characterized by abdominal tumours, with no specific geographic or climatic distribution 6. It tends to arise in the lymphoid tissues of the gut and the upper respiratory tract, often presenting as masses in the Waldeyer ring or the terminal ileum, or even with massive abdominal involvement. It could be associated with EBV in approximately 30% of cases.

Symptoms of sporadic BL are usually aspecific: abdominal pain, nausea, vomiting, bowel obstruction, gastrointestinal bleeding have been reported. Bowel or mesenteric lymphonodes are frequent intra-abdominal localizations, but also kidney, pancreas, liver, spleen, breast, or ovarian involvements can occur. At diagnosis, patients may have bulky disease and elevated levels of lactate dehydrogenase and uric acid. Involvement of bone marrow and central nervous system (CNS) is reported in 30–38% and 13–17% of adults, respectively 7–9. Bone marrow involvement is more comm seen in progressive disease. Iin fact Burkitt’s leukemia is essentially considered a presentation of advanced stage of BL, and it includes patients with acute lymphoblastic leukemia (1–2%), and circulating blasts that morphologically and histologically resemble BL’s cells.

## Treatment:

Before the advent of high intensity chemotherapy, BL was associated with poor outcomes, probably because of its high proliferative rate. The introduction of high intensity regimens has significantly changed the prognosis of this disease. At present BL appears to be curable in a high proportion of cases, when treated with aggressive multiagent- based chemotherapy regimens.

## Evolution of treatment :

The optimal for BL evolved over the last years, with the growing knowledge of the biological characteristics of the disease, usc as the rapid double time of the tumor, the propensity for extranodal sites, the high chemosensitivity, and the potential for CNS relapses.

Characteristic of BL is the chemo-sensitivity to single agents 1–3,6. In fact, using cyclophosphamide (CTX) alone, complete response (CR) rates of more than 70% 3 were obtained; however the high rates of relapse after such treatment have induced to employ treatment with cyclical CTX, followed by two cycles of combination therapy with vincristine (VCR), methotrexate (MTX) and/or cytosine arabinoside (Ara-C) in the event of relapsed disease. Despite the good results in patients with limited disease (up to 90% survival at 100 weeks), most of patients with CNS or bone marrow involvement relapsed and died 3,6.

Chemotherapy approaches in adults (Table 1) have been largely adapted from pediatric regimens 7–9. The main regimens for the front-line therapy of BL are reported in Table 2.

In 1995 and 1996, two reports documented the success of adapting the pediatric French LMB and the German BFM regimens to the treatment of adult BL 9,11. In a retrospective review 65 adults were treated according to the pediatric LMB 81, 84, 86, and 89 regimens, consisting of an initial cytoreductive phase, using CTX and prednisone to reduce the tumour burden and to minimize the risk of tumor lysis, followed by 2 induction cycles, 1 to 2 consolidation cycles, and 1 to 4 maintenance cycles. In these series 58 patients (89%) achieved a CR with a 3-year OS of 74%, even if most patients had advanced-stage disease or evidence of leukemic involvement; 7 of 12 patients who presented with CNS involvement remained disease free up to 56 months after therapy 9. A prospective study of the LMB protocol in adults confirmed the retrospective findings, with a CR rate of 83% and a 2-year OS of 66% 12.

After the success in pediatric BL with the BFM protocols, the German Multicenter Study Group for the treatment of adult ALL (GMALL) developed 2 protocols, B-NHL 83 and B-NHL 86, for the treatment of adult Burkitt’s leukemia 11. Similarly to the LMB trials, these studies included a cytoreductive pre-phase to minimize the risk of tumour lysis, after which 6 cycles of alternating chemotherapy regimens were given, with fractionated CTX, MTX, and low-dose Ara-C in each of these alternating cycles. In the B-NHL 86 regimen the dose of MTX was escalated to 1500 mg/m^2^ and ifosfamide was added to the B-NHL 83 regimen. Results were comparable to those noted in the French LMB trials, with 4- to 8-year OS reaching 49–51% 11.

McMaster *et al* treated 20 patients with 2 intensive inpatient induction courses of high-dose CTX, MTX (200 mg/m^2^), bleomycin, VCR, and doxorubicin achieving CR in 85% of patients, with 5-year DFS of 60% 8. The Stanford group obtained similar results with a regimen containing high-dose CTX (1500 mg/m^2^) and mid-cycle high-dose MTX (3000 mg/m^2^) administered over 6 to 9 cycles. With this regimen, 2-year OS reached 66.8%; however, the best responses were noted in patients with limited-stage disease (a single extra-abdominal tumour site or a completely resected intra-abdominal disease), where 2-year OS was 100%, compared with 53.8% in the advanced setting 13.

The experiences with dose-dense regimens culminated in the scheme reported by Magrath and colleagues 10. Pediatric and young adult patients with not-cleaved small lymphocitic lymphomas, when high risk, were treated alternating cycles with Cyclophosfamide (CTX), Vincristine (VCR), doxorubicin, high-dose Methotrexate (MTX)i.v. and intrathecal (CODOX-M) and cicles including ifosfamide, etoposide, high-dose Ara-C i.v. and intrathecal (IVAC regimen), and when low risk received three cycles of CTX, VCR, doxorubicin and high-dose MTX (CODOX-M) 10. This study demonstrated that short courses of intensive therapy had excellent response rates, with 92% 2-year EFS both for children and adults. Unfortunately, the toxicities reported from many of these intensive regimens are significant, including neurotoxicities from intrathecal therapy, hematological toxicity and severe mucositis.). A subsequent international validation of this first impressing data, obteined in children and young adults, came from an international cohort of more aged patients 7.

## The current approaches to Burkitt ’s lymphoma:

Over the past few years, more focus has been placed on identifying efficacious but less toxic regimens. Several trials aimed at reducing MTX-associated toxicity, while maintaining the treatment’s efficacy. Utilizing a modified Magrath’s regimen, one small study treated adults with reduced doses of systemic MTX and intrathecal Ara-C, and altered the fractionated schedule for the CTX. This resulted in a significant decrease in neurotoxicity and mucositis, and there were no treatment-associated deaths. Overall, the 2-year EFS was 64%, but 100% for low-risk patients (normal LDH and focal, smaller volume tumour burden) and 60% for high-risk patients 14. This study proved that some patients can be effectively treated with lower intensity regimens, with reduced drug toxicity. The inferior survival of high-risk patients may reflect the change in regimen, but it may also be explained by other factors, different in the two studies. For example, the median age was 46 years in the latter study, compared with 24 years for the original study by Magrath *et al*. It has became increasingly clear that age is a significant prognostic factor related to survival, as older patients do not tolerate the chemotherapy as well as the younger, or do not have the same good response. This fact could be explained by the innate differences in the biology of the tumours. The results of a FAB/LMB96 trial for pediatric intermediate-risk NHL patients revealed that it is possible to reduce treatment for early responding patients. In this trial, the intermediate-risk patients (defined as non-resected, stage I/II and CNS negative advanced-stage III/IV), who have had an early response to therapy (> 20% response at day 7), could be treated with reduced doses of CTX and doxorubicin without a significant decrement in EFS and in overall survival, comparing to the original trial. This approach should reduce the risk of future toxicities such as cardiac disease or infertility 15,16. With the Hyper-CVAD regimen, a modified Murphy regimen used to treat adult Burkitt leukemia at MD Anderson, 81% of patients achieved a CR, with a 3-year OS of 49%. Notably, this study contained a much older population of patients (median age, 58 years) than that reported in other trials, and patients 60 years or older had an inferior outcome (3-year OS of 17% versus 77%) 17. The CALGB regimen contains a cytoreductive phase, followed by 3 cycles, each of 2 different regimens administered every 3 weeks [18]. In 54 evaluable patients, CRs were noted in 80%, with 4-year DFS of 50%. However, severe neurologic toxicity was observed in 10 of 74 patients enrolled on this trial, attributed to the combination of high-dose MTX (1500 mg/m2), triple intrathecal chemotherapy, and whole brain irradiation (24 Gy) used for CNS prophylaxis. The cranial radiation was subsequently eliminated for patients without bone marrow involvement at presentation and the rate of neurologic events decreased. In the CALGB study, 32% of patients older than 50 years were able to complete 6 to 7 cycles of treatment, compared with 79% of younger patients .Mortaliy (21% versus 9%) disease progression (32% versus 3%), and toxicity (16% versus 9%) were noted to be higher in those patients older than 50 years 8. The higher rate of relapse in elderly patients with BL implies that these poor outcomes may not simply be related to treatment-related toxicity.

Thomas et al noted an increased incidence of complex cytogenetic abnormalities in older patients, including bcl-2 gene rearrangements, which may contribute to a more aggressive phenotype 19. A prospective study of dose-modified CODOX-M/IVAC was recently conducted in patients with sporadic BL, defined using cytogenetic and immunophenotypic criteria; immunophenotype and fluorescent in situ hybridization (FISH) were used to separate BL from other aggressive B-cell lymphomas. Compared with the previous trial LY06 with full-dose MTX (6.7 g/m(2) 5, there was a reduction in toxicity with comparable outcomes in patients treated dose-modified CODOX-M (MTX, dose 3 g/m^2^) with or without IVAC, according to risk group 20.

## Monoclonal antibodies and new drugs:

It seems interesting the use of monoclonal antibodies and other biological reagents as adjuvant therapy in BL. These include agents such as anti-CD20 monoclonal antibody (Rituximab). It has been used most extensively with CTX, VCR, doxorubicin and dexamethasone [17,21] or with ifosfamide, carboplatin, etoposide in children with refractory BL 22.

A recent study of 31 patients treated with hyper-fractionated CTX, VCR, doxorubicin, and dexamethasone (hyper-CVAD) regimen plus rituximab demonstrated a significant increase in overall survival, EFS and disease-free survival (89, 80 and 88%, respectively), when compared with historical patients treated with CTX, VCR, doxorubicin and dexamethasone alone 17.

Interestingly, preliminary results of 19 patients (median age 29 years, with 53% advanced stage III/IV) with BL treated using dose-adjusted etoposide, prednisone, VCR, CTX and doxorubicin and rituximab (a known effective therapeutic regimen for the treatment of DLBCL); remission was obtained in all patients, without any relapses reported, with an average follow-up of 28 months. In addition, the therapy was administered in an outpatient, on the basis of the minimal side effects reported 21.

Other biological agents include monoclonal antibodies such as anti-CD22 (epratuzumab), in study for the treatment of NHL. Respect to rituximab, it has a different mechanism of action, but they could be synergistic in inducing cellular apoptosis 23.

Novel treatment options are developed basing on the rapidly growing knowledge about the molecular biology of this disease. In early development are epigenetic regulators such as histone deacetylases inhibitors and DNA methyltransferases inhibitors. Among them, Depsipeptide is a histone deacetylases inhibitor under investigation, because it has been shown to have an additive cytotoxic effect with many of the standard chemotherapies used for the treatment of lymphomas and leukemias (including BL) 24.

Other agents can theoretically be used to target oncogenes, such as small peptide nucleic acids. A recent study showed that BL developing in severe combined immunodeficient mice can be inhibited by a peptide nucleic acid complementary to regulatory intronic sequences, reducing *c-myc* production. Boffa and colleagues demonstrated a significant reduction in tumour size and progression of disease after the use of small peptide nucleic acids complementary to a regulatory sequence for *c-myc* 25,26.

## Hemopoietic stem cell transplantation:

The role of stem cell transplantation in the treatment of BL has been explored over the past 12 years. Several studies have focused on the potential benefit of high-dose chemotherapy followed by autologous stem cell transplantation, with some promising results in response rates and overall survival 27,28. A recent phase II study for adult patients by van Imhoff *et al* 29 utilized an up-front short intensive chemotherapy course followed by autologous stem cell transplantation: this scheme resulted in equivalent, or slightly better, 5-year EFS and overall survival compared with current chemotherapy regimens. One of the limitations for the study was the low number of patients with bone marrow involvement, compared with other studies utilizing intensive chemotherapy. It has been well documented that bone marrow involvement is a known poor prognostic indicator for BL 30,31.

In addition, there have been reports of retrospective evaluations of allogeneic transplantation for BL. The theoretical benefits include the removal of the possibility of tumour contamination, and the still controverse graft versus lymphoma effect. Such reports show lower relapse rates for patients receiving allogeneic transplantation when compared with recipients of autologous transplantation, but unfortunately with higher rates of transplant-related mortality 32. At the same time, there have been case-reports of allogeneic transplantations leading to complete response and long survival of individual patients 33,34. The existence of a significant graft versus lymphoma effect from allogeneic stem cell transplantation is still widely debated 33,35.

At this time the role of hemopoietic stem cell transplant for BL is difficult to be defined.

## Conclusions:

The behaviour of BL to chemotherapy approaches represents a very interesting point. In the past it was thought to be an incurable disease in adults because of its high proliferative rates, but the incorporation in treatment regimens of several active agents, particularly of the cyclophosphamide and the high-dose methotrexate, improved the outcome in these patients, reaching a very high cure rate. A maintenance phase does not seem to add an amelioration of outcome, while the prognosis of patients has further improved after the addition of rituximab to chemotherapy regimens. The use of hemopoietic stem cell transplantation remains experimental, and it should be reserved for younger patients with refractory or resistant disease.

## Figures and Tables

**Table 1.** Results of treatment of adult sporadic BL.

**REFERENCE**	**Protocol**	**No. of patients**	**Median age, y (range)**	**CR, %**	**DFS, %**	**EFS, %**	**OS, %**
**Bernstein*et al*11**	Stanford	18	25 (15–75)	78	71.3 at 1 y	N/A	66.8 at 2 y
**Lopez*et al*6**	MD Anderson 81–01 and 84–30	44	32 (17–72)	80	60 at 5 y	N/A	52 at 5 y
**McMaster*et al*29**	Vanderbilt	20	44.5 (21–69)	85	65 at 5 y	N/A	N/A
**Divinè*et al*28**	ACVBP	52	34	85	N/A	47 at 5 y	53 at 5 y
**Soussain*et al*7**	LMB 81, 84, 86 and 89	65	26 (17–65)	89	N/A	71 at 3 y	74 at 3 y
**Hoeltzer*et al*9**	BNHL83	24	33 (15–38)	63	50 at 8 y	N/A	49 at 8 y
**Hoeltzer*et al*9**	BNHL86	35	36 (18–65)	74	71 at 4 y	N/A	51 at 4 y
**LaCasce*et al*12**	CODOX-M/IVAC	14	47	86	72 at 21 mo	N/A	N/A
**Mead*et al*5**	CODOX-M/IVAC	52	35 (15–60)	75	N/A	64.6 at 2 y	72.8 at 2 y
**Thomas*et al*17**	Hyper-CVAD	26	58 (17–79)	81	61 at 3 y	N/A	49 at 3 y
**Lee*et al*16**	CALGB 9251	54	44 (18–71)	80	50 at 4 y	N/A	52 at 4 y

CR: complete remission; DFS: disease free survival; EFS: even- free survival; OS: overall survival

**Table 2.** Main front-line specific chemotherapy regimens used in the treatment of BL.

**REGIMEN**	**SCHEDULE**
**STANFORD**	- Cyclophosphamide 1200 mg/m2 day 1 - Doxorubicin 40 mg/m2 day 1 - Vincristine 1.4 mg/m2 (maximum 2 mg) day 1 - Prednisone 40 mg/m2 days 1–5 - Methotrexate 3000 mg/m2 (with leucovorin rescue) day 10 - IT Methotrexate 12 mg days 1 and 10
**LMB 84, 86, and 89**	**Cytoreductive phase (COP)** - Cyclophosphamide 300 mg/m2 day 1 - Vincristine 2 mg day 1 - Prednisone 60 mg/m2/day, days 1–7 - IT Methotrexate and Hydrocortisone day 1 **Induction (COPADM1)** - Cyclophosphamide 500 mg/m2/day, days 2–4 - Doxorubicin 60 mg/m2 day 2 - Vincristine 2 mg day 1 - Methotrexate 3000 – 8000 mg/m2 over 3 hrs day 1 (with leucovorin) - Prednisone 60 mg/m2/day, days 1–7 - IT Methotrexate and Hydrocortisone days 2 and 8 **Induction (COPADM2)** - Cyclophosphamide 1000 mg/m2/day, days 2–4 - Doxorubicin 60 mg/m2 day 2 - Vincristine 2 mg days 1 and 6 - Methotrexate 3000 – 8000 mg/m2 over 3 hrs day 1 (with leucovorin) - Prednisone 60 mg/m2/day, days 1–7 - IT Methotrexate and Hydrocortisone days 2 and 8 **Consolidation x 2** - Etoposide 200 mg/m2 (LMB 86 only) - Methotrexate 3000 mg/m2 over 3 hrs day 1 (with leucovorin) - Cytarabine 100 mg/m2/day days 1–5 (LMB 84) or 3000 mg/m2/day - days 2–5 (LMB 86) - IT Methotrexate and Hydrocortisone day 2, IT Cytarabine and - Hydrocortisone day 7 **Maintenance (1–4 cycles)** - Cyclophosphamide 500 mg/m2/day, days 1–2 - Doxorubicin 60 mg/m2 day 2 - Vincristine 2 mg day 1 - Methotrexate 3000 mg/m2 over 3 hrs day 1 (with leucovorin) - Prednisone 60 mg/m2/day, days 1–5 - IT Methotrexate and Hydrocortisone day 2
**BNHL-86: Prephase, followed by alternating A/B cycles for 6 cycles**	**Prephase** - Cyclophosphamide 200 mg/m2/day, days 1–5 - Prednisone 60 mg/m2/day, days 1–5 **Cycle A** - Ifosfamide 800 mg/m2/day, days 1–5 - VM26 100 mg/m2/day, days 4 and 5 - Vincristine 2 mg day 1 - Cytarabine 150 mg/m2 q12 hrs x 4 doses, days 4 and 5 - Methotrexate 1500mg/m2 over 24 hours day 1 (with leucovorin) - Dexamethasone 10 mg/m2/day, days 1–5 - IT Methotrexate 15 mg, IT Cytarabine 40 mg, IT Dexamethasone 4 mg, days 1 and 5 **Cycle B** - Cyclophosphamide 200 mg/m2/day, days 1–5 - Doxorubicin 25 mg/m2/day, days 4 and 5 - Vincristine 2mg IV day 1 - Methotrexate 1500 mg/m2 over 24 hours day 1 (with leucovorin) - Dexamethasone 10 mg/m2/day, days 1–5 - IT Methotrexate 15 mg, IT Cytarabine 40 mg, IT dexamethasone 4 mg, day 1
**CODOX-M/IVAC: Alternate CODOX-M/IVAC cycles for 4 cycles**	**CODOX-M** - Cyclophosphamide 800 mg/m2 day 1 and 200 mg/m2/day, days 2–5 - Doxorubicin 40 mg/m2/day, day 1 - Vincristine 1.5 mg/m2/day, days 1 and 8 - Methotrexate 1200 mg/m2 over 1 h and then 240 mg/m2/hr for 23 hr (with leucovorin) day 10 - IT Cytarabine 70 mg days 1 and 3, IT Methotrexate 12 mg day 15 **IVAC** - Ifosfamide 1500 mg/m2/day days 1–5 (with mesna) - Etoposide 60 mg/m2/day, days 1–5 - Cytarabine 2000 mg/m2 every 12 hours for 4 doses, days 1 and 2 - IT Methotrexate 12 mg day 5
**Hyper-CVAD: Alternate cycles 1 and 2 for 8 cycles**	**Cycle 1** - Cyclophosphamide 300 mg/m2 q12 hours x 6 doses, days 1–3 (with mesna) - Doxorubicin 50 mg/m2 day 4 - Vincristine 2 mg/day, days 4 and 11 - Dexamethasone 40 mg/day, days 1–4 and 11–14 - IT Methotrexate 12 mg day 2 and IT Cytarabine 100 mg day 7 **Cycle 2** - Methotrexate 1000 mg/m2 day 1 (with leucovorin rescue) - Cytarabine 3000 mg/m2 q12 hours x 4 doses, days 2 and 3 - IT Methotrexate 12 mg day 2 and IT Cytarabine 100 mg day 7
**CALGB 9251: Prephase, followed by alternating cycles 2–7**	**Prephase** - Cyclophosphamide 200 mg/m2/day, days 1–5 - Prednisone 60 mg/m2/day, days 1–5 **Cycles 2, 4, and 6** - Ifosfamide 800 mg/m2/day, days 1–5 - Mesna 200 mg/m2/day, at 0, 4, and 8 hours after ifosfamide, days 1–5 - Vincristine 2 mg day 1 - Etoposide 80 mg/m2/days, days 4 and 5 - Cytarabine 150 mg/m2/day continuous infusion, days 4 and 5 - Methotrexate 150 mg/m2 over 30 min, then 1.35 g/m2 over 23.5 hrs, day 1 (with leucovorin rescue) - Dexamethasone 10 mg/m2/day, days 1–5 - IT Methotrexate 15 mg, IT Cytarabine 40 mg, IT hydrocortisone 50 mg, days 1 and 5 **Cycles 3, 5, and 7** - Cyclophosphamide 200 mg/m2/day, days 1–5 - Doxorubicin 25 mg/m2/day, days 4 and 5 - Vincristine 2mg IV day 1 - Methotrexate 150 mg/m2 over 30 min, then 1.35 g/m2 over 23.5 hrs, day 1 (with leucovorin rescue) - Dexamethasone 10 mg/m2/day, days 1–5 - IT Methotrexate 15 mg, IT Cytarabine 40 mg, IT hydrocortisone 50 mg, days 1 and 5 * * Cranial irradiation 24 Gy in12 fractions after cycle 3
